# Downregulation of *RCN1* inhibits esophageal squamous cell carcinoma progression and M2 macrophage polarization

**DOI:** 10.1371/journal.pone.0302780

**Published:** 2024-05-07

**Authors:** Haiyang Guo, Jinghao Shu, Guangbing Hu, Bingyang Liu, Jie Li, Jinhong Sun, Xiaobo Wang, Han Liu, Shiyu Xiong, Yong Tang, Yaolin Yin, Xianfei Wang

**Affiliations:** 1 Department of Gastroenterology, Affiliated Hospital of North Sichuan Medical College, Nanchong, Sichuan, China; 2 Digestive Endoscopy Center, Affiliated Hospital of North Sichuan Medical College, Nanchong, Sichuan, China; Shantou University Medical College, CHINA

## Abstract

Reticulocalbin 1 (RCN1) is a calcium-binding protein involved in the regulation of calcium homeostasis in the endoplasmic reticulum. The aim of this study was to explore the clinical value and biological role of *RCN1* in esophageal squamous cell carcinoma (ESCC). In addition, we investigated the effect of RCN1 on the polarization of tumor-associated macrophages (TAMs). The GSE53625 dataset from the Gene Expression Omnibus database was used to analyze the expression of *RCN1* mRNA and its relationship with clinical value and immune cell infiltration. Immunohistochemistry was used to validate the expression of RCN1 and its correlation with clinicopathological characteristics. Subsequently, transwell and cell scratch assays were conducted to evaluate the migration and invasion abilities of ESCC cells. The expression levels of epithelial–mesenchymal transition (EMT)-related proteins were evaluated by western blot, while apoptosis was detected by flow cytometry and western blot. Additionally, qRT‒PCR was utilized to evaluate the role of RCN1 in macrophage polarization. *RCN1* was significantly upregulated in ESCC tissues and was closely associated with lymphatic metastasis and a poor prognosis, and was an independent prognostic factor for ESCC in patients. Knockdown of *RCN1* significantly inhibited the migration, invasion, and EMT of ESCC cells, and promoted cell apoptosis. In addition, RCN1 downregulation inhibited M2 polarization. *RCN1* is upregulated in ESCC patients and is negatively correlated with patient prognosis. Knocking down *RCN1* inhibits ESCC progression and M2 polarization. *RCN1* can serve as a potential diagnostic and prognostic indicator for ESCC, and targeting RCN1 is a very promising therapeutic strategy.

## Introduction

Esophageal cancer is one of the most common malignancies worldwide and is the sixth leading cause of cancer-related death [1]. The annual incidence and mortality rates of esophageal cancer in China comprise more than half of total global cases, with esophageal squamous cell carcinoma (ESCC) being the most common subtype, accounting for more than 90% of cases [2]. Due to the atypical early symptoms of ESCC, the lack of effective diagnostic and treatment targets, and the fact that most patients have already developed metastasis at the time of diagnosis, the 5-year survival rate is less than 30% [3]. Therefore, further research on the pathogenesis of esophageal cancer is of great significance to identifying new therapeutic targets and prognostic indicators to improve the prognosis of patients with esophageal cancer.

Reticulocalbin 1 (RCN1) is a member of the CREC protein family; it is a calcium-binding protein that resides in the endoplasmic reticulum (ER) and is involved in regulating calcium-dependent activity in the ER lumen [4]. RCN1 is not only confined to the ER lumen but also found on the surfaces of cells, with its expression being heterogeneous across various cell types [5]. There have been reports of RCN1 protein dysregulation in a variety of diseases, including cardiovascular disease [6], neuromuscular disease [7], and [8] tumors. In recent years, *RCN1* has received widespread attention due to its important role in tumorigenesis and progression [9–13]. For example, in prostate cancer cells, downregulation of *RCN1* promoted apoptosis and necrosis of the cells [13]; in non-small cell lung cancer, high expression of the RCN1 protein was found to be significantly associated with poor prognosis in non-small cell lung cancer patients, and inhibition of RCN1 was shown to reduce proliferation, migration, and invasion of lung adenocarcinoma cells [12]. These findings suggest that *RCN1* may play a role as an oncogene in cancer. However, the expression pattern and biological behavior of *RCN1* in ESCC are not yet clear.

Tumor-associated macrophages (TAMs) are macrophages that exist in the microenvironment of solid tumors and are the most common immune-related cells in the tumor microenvironment. TAMs manifest mainly as two types of macrophages: classically activated macrophages (M1) and alternatively activated macrophages (M2). M1 macrophages have antitumor effects, while M2 macrophages promote tumor progression [14]. Previous studies have also shown that a high density of M2 macrophages is associated with poor prognosis in patients with ESCC [15, 16]. However, it is not clear whether RCN1 regulates TAM infiltration and polarization.

In our study, we first utilized the GSE53625 dataset to evaluate the expression and prognostic significance of *RCN1* in ESCC. Subsequently, we validated the protein expression of RCN1 in ESCC and paracancerous tissues by immunohistochemistry (IHC) and analyzed the relationship between RCN1 and clinicopathological characteristics in ESCC patients. We then evaluated the effects of *RCN1* knockdown on the migration, invasion, epithelial–mesenchymal transition (EMT), and apoptosis of ESCC cells. Additionally, we explored the regulatory role of *RCN1* in macrophage polarization.

## Materials and methods

### Collection of the dataset

We downloaded the GSE53625 dataset [17] from the Gene Expression Omnibus (GEO) database (https://www.ncbi.nlm.nih.gov/geo/) on December 1, 2022, and then used the "limma" package [18] to remove the batch effects. The GSE53625 dataset is the largest ESCC dataset available in the public database, containing 179 pairs of ESCC and paraneoplastic tissues. We extracted the *RCN1* mRNA expression matrix and its corresponding clinical data from the GSE53625 dataset for further analysis.

### Correlation analysis between *RCN1* mRNA expression and clinicopathological characteristics

We analyzed the expression level of *RCN1* in ESCC and paracancerous tissues using the "limma" R package from the GSE53625 dataset. We validated the expression of *RCN1* in The Cancer Genome Atlas (TCGA) and Genotype-Tissue Expression (GTEx) databases using the GEPIA online analysis tool (http://gepia.cancer-pku.cn) [19]. We used the "ggpubr" R package to analyze the correlation between the expression level of *RCN1* mRNA and the clinical pathological characteristics of ESCC patients.

### Diagnosis and prognostic analysis of *RCN1* mRNA

ESCC patients were divided into high- and low-expression groups based on the median value of *RCN1* mRNA expression. The Kaplan–Meier method was used to analyze and evaluate the relationship between the expression of *RCN1* and the prognosis of ESCC patients. The "pROC" R software package was used to evaluate the diagnostic value of *RCN1* in ESCC. The "survival" package was used to perform univariate and multivariate Cox regression analyses to explore independent prognostic factors for ESCC patients. Subsequently, based on the results of a multifactorial Cox regression analysis, we constructed a nomogram to predict the survival of ESCC patients at 1, 3, and 5 years. Furthermore, we plotted a calibration chart to evaluate the accuracy of the nomogram.

### Analysis of the correlation between *RCN1* mRNA and immune cell infiltration

To evaluate the correlation between *RCN1* and immune cell infiltration, we first used Cell-type Identification by Estimating Relative Subsets of RNA Transcripts (CIBERSORT) [20] to analyze the infiltration of immune cells in each patient in the GSE53625 dataset. Then, we used the Wilcoxon signed rank-sum test to compare the differences in 22 types of immune cells between the high and low *RCN1* expression groups.

### Collection of clinical samples

On December 15, 2022, we collected paraffin sections of cancer and paracarcinoma tissues along with clinical information from 80 ESCC patients who had surgery at the Affiliated Hospital of North Sichuan Medical College between January 2018 and December 2020. None of the patients received any chemoradiotherapy or other antitumor drug therapy before surgery. These 80 patients had complete clinical data, and their baseline data are presented in S1 Table. The pathological sections were confirmed as ESCC by two experienced pathologists. During or after the data collection, we did not obtain any information that could identify individual participants. This study was approved by the Medical Ethics Committee of the Affiliated Hospital of North Sichuan Medical College (Approval number: 2022ER441-1) and was conducted in accordance with the Helsinki Declaration. Since this study is a retrospective study and all patient data are anonymous, informed consent was waived with the approval of the Medical Ethics Committee of the Affiliated Hospital of North Sichuan Medical College.

### Immunohistochemistry

IHC analysis was used to examine the protein expression of RCN1 in ESCC tissues and paracancerous tissues. After deparaffinization of the paraffin sections, gradient rehydration was performed, followed by antigen retrieval using the high-pressure steam method. After removing endogenous peroxidases with 3% hydrogen peroxide, the sections were blocked with normal goat serum. Subsequently, the RCN1 antibody (Ab210404, Abcam) was added to the sections at a 1:200 dilution and incubated overnight at 4°C. Then, the sections were incubated with the appropriate secondary antibody, detected with DAB as the chromogenic substrate, counterstained with hematoxylin, dehydrated in graded ethanol, and mounted with neutral resin. The immune reactivity score (IRS) was calculated as the product of the percentage of stained cells (0: unstained; 1: ≤10%; 2: 11–50%; 3: 51–80%; 4: ≥81%) and the staining intensity (0: no staining intensity; 1: weak; 2: moderate; 4: strong).

### Cell culture and transfection

ESCC cell lines (KYSE150, KYSE510, KYSE30, and KYSE410) and the human monocyte cell line THP-1 were purchased from Cellcook (Guangzhou, China). All cell lines were cultured in RPMI-1640 medium containing 10% fetal bovine serum and were maintained in a humidified atmosphere with 5% CO_2_ at 37°C. To knock down endogenous *RCN1* expression, the short hairpin RNA (shRNA) sequences were cloned and inserted into the hU6-MCS-CBh-gcGFP-IRES-puromycin vector and used to infect the KYSE150 and KYSE410 cell lines with an MOI (multiplicity of infection) value of 10. Lentiviral vectors were synthesized by Genechem (Shanghai, China). The sequences used for knockdown of *RCN1* expression were shRCN1#1, AGAAGCTAACTAAAGAGGAAA; shRCN1#2, GACGGGAAGTTAGACAAAGAT; shRCN1#3, CATCTTTGATAATGTCGCCAA; and the negative control (NC) sequence was shNC, TTCTCCGAACGTGTCACGT.

### Quantitative real-time PCR (qRT‒PCR)

Total RNA was extracted from the cells using TRIzol reagent. HiScript III RT SuperMix for qPCR (Vazyme Biotech Co., Ltd., China) was used for qRT‒PCRs on the LightCycler® 480 system (Roche Diagnostics, Risch, Switzerland). GAPDH was used as an internal reference gene. The primers used are shown in S2 Table.

### Western blot

Cellular proteins were extracted using RIPA buffer (Appygen, Beijing, China) containing phosphatase inhibitors. Proteins were separated on a 10% SDS–PAGE gel and then transferred to a PVDF membrane (Sigma–Aldrich, Missouri, USA). The membrane was blocked with 5% skim milk for 2 h and then incubated with the primary antibody overnight at 4°C. After incubation with the appropriate secondary antibody for 1 h, the bands were detected using the ChemiDoc™ XRS+ system (Bio-Rad, California, USA). The gray values of protein bands were determined using ImageJ software, and the relative protein expression was calculated by normalizing to GAPDH as an internal reference. Western blot was repeated three times for each protein. Detailed information for all the antibodies is shown in S3 Table.

### Cell scratch experiment

Cells were seeded in a 6-well plate and cultured until they reached 90% confluency. The cell layer was then scratched using a 200 μl pipette tip and washed with serum-free RPMI 1640 medium. Pictures were taken at 0 and 24 h in the same position.

### Migration and invasion assays

Cell migration and invasion abilities were detected using transwell chambers with an 8-μm pore size (Corning, USA). For cell migration, 5 × 10^4^ cells were seeded in the upper chamber, and 600 μl of medium containing 10% FBS was added to the lower chamber. After 48 h, the cells were fixed with 4% paraformaldehyde and stained with 0.1% crystal violet. Images were captured using an inverted microscope. For cell invasion, a layer of basement membrane matrix was coated in the upper chamber before seeding 5 × 10^4^ cells. All other steps were the same as those in the cell migration experiment. Cell numbers were quantified using ImageJ software.

### Flow cytometry

Cell apoptosis was detected using the Annexin V-APC/propidium iodide (PI) apoptosis detection kit (KeyGen Biotech Co., Ltd., Nanjing, China) according to the manufacturer’s instructions. In brief, we seeded 5 × 10^5^ cells in a 60mm culture dish and collected 5 × 10^5^ cells after 24 h using trypsin, followed by three washes with PBS. We then resuspended the cells in 500 μl of binding buffer, added 5 μl of Annexin V-APC, and added 5 μl of PI. After incubating in the dark for 10 min, flow cytometry (ACEA Bioscience, USA) analysis was performed, with 2–4×10^4^ cells analyzed in each run.

### Co-culture experiments with THP-1 cells

THP-1 cells were seeded in 6-well plates and induced with 100 ng/mL phorbol-12-myristate-13-acetate (PMA) for 24 h to differentiate into M0 macrophages. A cell culture insert (0.4-μm pores; Corning, USA) was placed in a 6-well plate, and ESCC cells were seeded in the insert. After co-culture for 48 h, macrophages were collected for mRNA and protein expression analysis.

### Statistical analysis

Data analysis and graphing were performed using R 4.0.2 software and GraphPad Prism 9 (GraphPad Software Inc., San Diego, CA, USA). Paired t tests and unpaired t tests were used for comparisons between two groups, and the one-way ANOVA followed by the Tukey post hoc test was used for comparisons among multiple groups. The chi-square test was used to analyze the correlation between *RCN1* expression and clinical pathological factors. Cox regression analysis was used to determine independent prognostic factors for ESCC patients. A *p* < 0.05 indicated statistical significance (*p < 0.05, **p < 0.01, ***p < 0.001).

## Results

### *RCN1* is highly expressed in ESCC tissues

**Fig 1:**
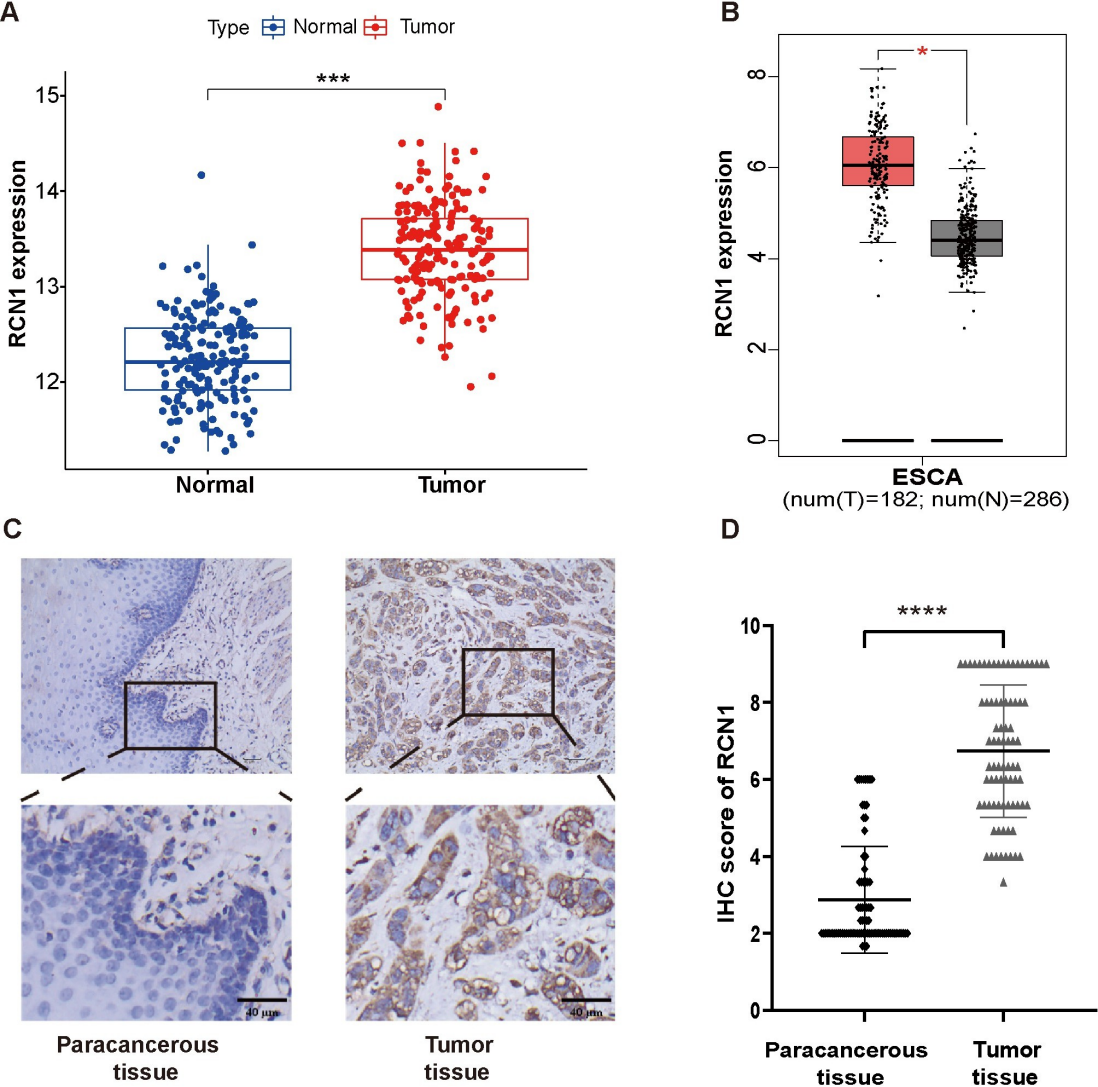
The expression level of *RCN1* in esophageal squamous cell carcinoma (ESCC) and paraneoplastic tissues. (A) Differential expression analysis of *RCN1* mRNA in ESCC and paracancerous tissues was performed using the GSE53625 dataset. (B) The mRNA expression levels of *RCN1* in ESCC and normal tissues were analyzed using the GEPIA database. (C) Representative immunohistochemical (IHC) images of RCN1 protein expression in ESCC and paraneoplastic tissues (original magnification, × 200). (D) Comparison of the protein expression levels of RCN1 in 80 pairs of ESCC and paraneoplastic tissues. *p <0.05, ***p* *<0.001, ****p <0.0001.

To elucidate the expression pattern of *RCN1* in ESCC tissues, we first analyzed the GSE53625 dataset and the GEPIA database. The results showed that *RCN1* mRNA was highly expressed in cancerous tissues (Fig 1A and 1B). Next, we also found that the protein expression level of RCN1 in ESCC tissues was higher than that in paracancerous tissues (Fig 1C and 1D). The above results indicate that the expression of *RCN1* is upregulated in ESCC tissues.

### *RCN1* is a potential diagnostic and prognostic marker for patients with ESCC

**Fig 2:**
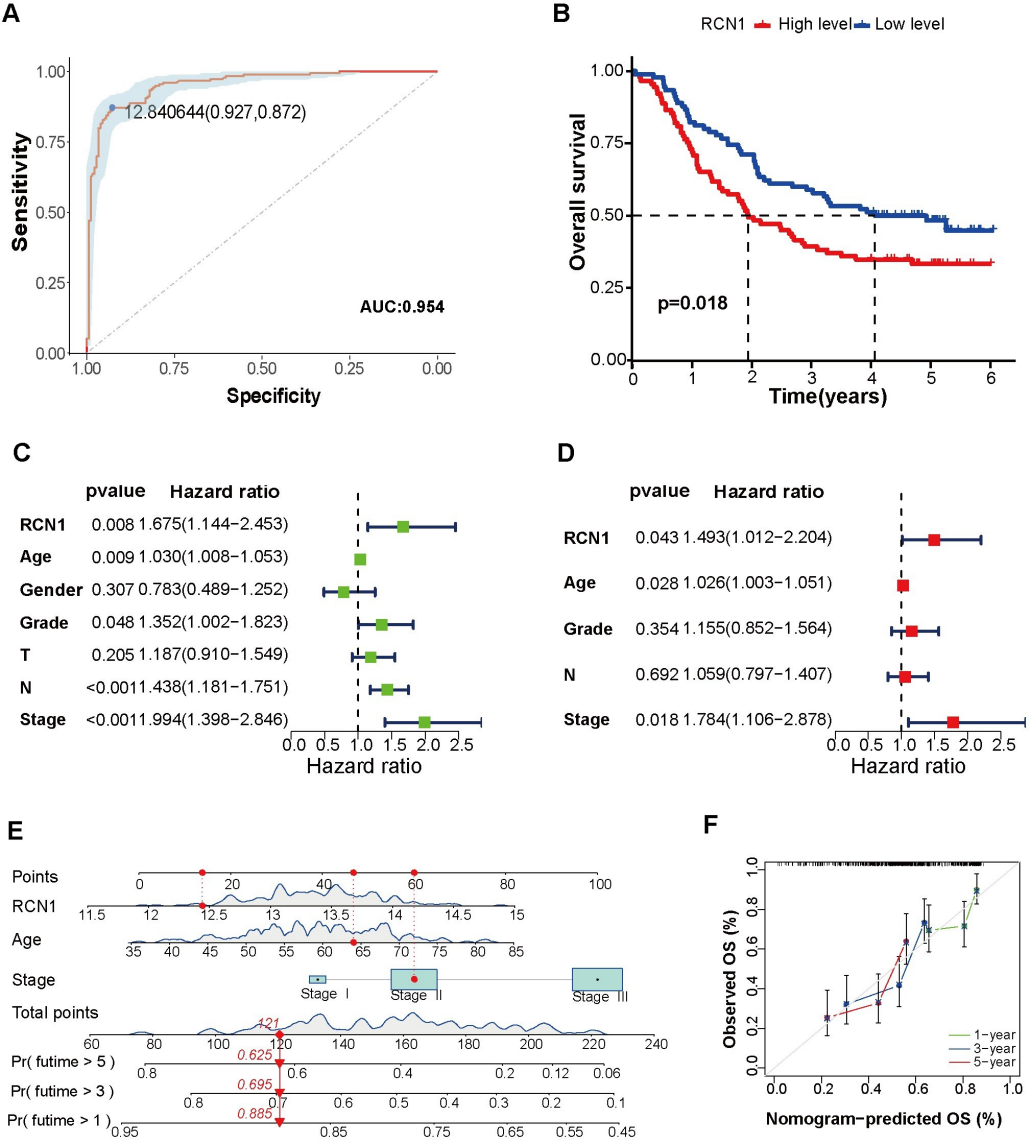
Diagnostic and prognostic value of *RCN1* in patients with esophageal squamous cell carcinoma (ESCC). (A) The diagnostic efficiency of *RCN1* for ESCC patients was assessed by plotting receiver operating characteristic (ROC) curves. (B) The relationship between *RCN1* expression and overall survival (OS) in ESCC patients was analyzed using Kaplan–Meier curves. (C and D) The prognostic risk factors for ESCC patients were analyzed using univariate and multifactorial Cox regression analyzes. (E) Plotting the nomogram predicts the overall survival of ESCC patients at 1, 3, and 5 years. (F) Calibration curve of the nomogram.

To investigate the clinical significance of *RCN1* expression levels, we utilized the GSE53625 dataset to evaluate the diagnostic and prognostic value of *RCN1* in ESCC patients. Receiver operating characteristic (ROC) curve analysis showed that the area under the curve (AUC) was 0.954 (Fig 2A), indicating that *RCN1* may be a potential diagnostic biomarker for ESCC patients. The Kaplan–Meier survival analysis showed that increased expression of *RCN1* was associated with a poor prognosis in ESCC patients (*p* = 0.018; Fig 2B). Univariate and multivariate Cox regression analyses revealed that *RCN1*, age, and stage were independent risk factors for ESCC patients (Fig 2C and 2D). Subsequently, a nomogram was constructed using *RCN1*, age, and stage to predict the 1, 3, and 5-year overall survival (OS) of ESCC patients (Fig 2E). The calibration curve showed that the nomogram had a certain predictive ability (Fig 2F). These results indicate that *RCN1* can serve as an important prognostic biomarker for ESCC patients.

### Relationship between RCN1 and the clinicopathological characteristics of ESCC patients

**Fig 3:**
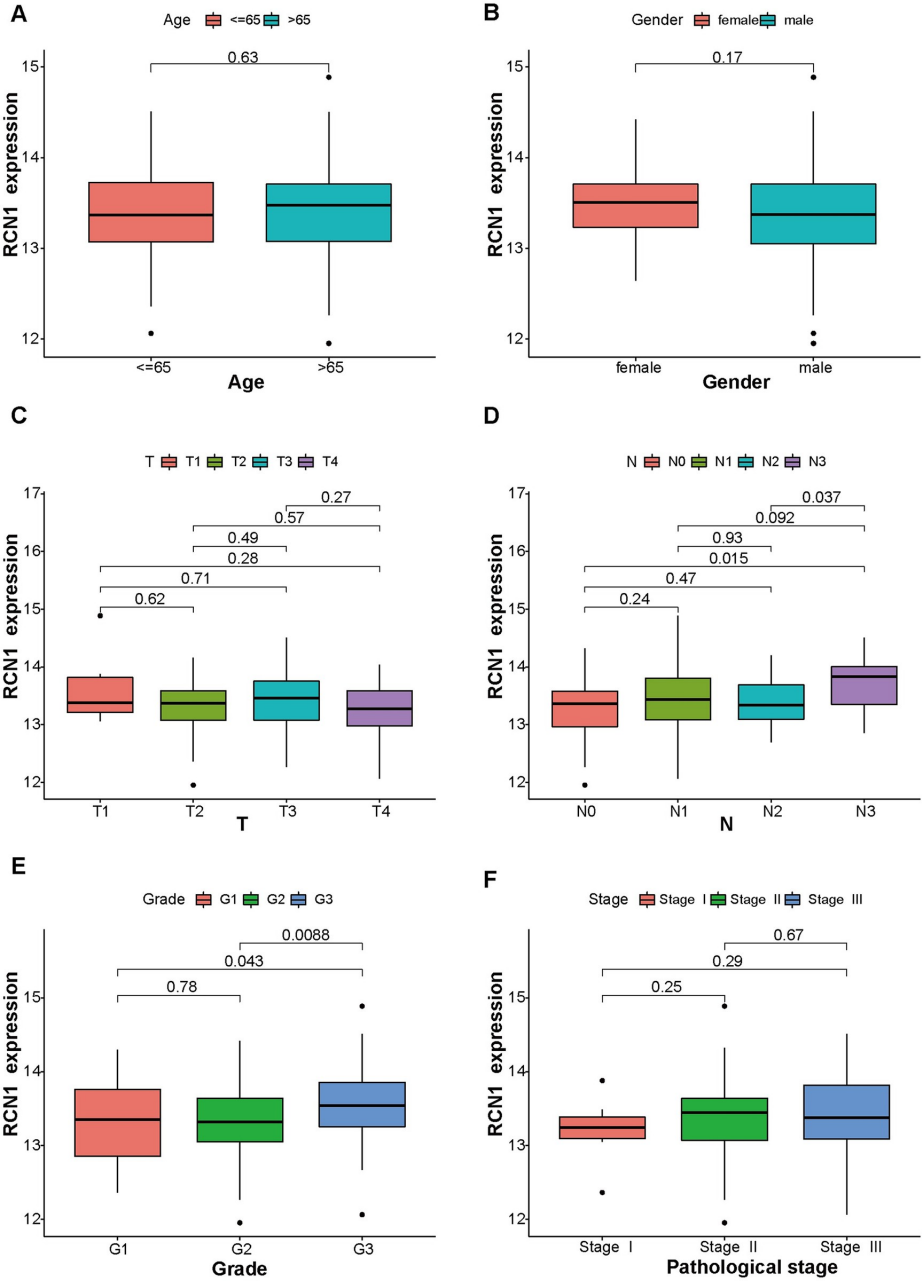
The relationship between *RCN1* mRNA expression and clinicopathological characteristics was analyzed using the GSE53625 dataset. (A) Age. (B) Gender. (C) T stage. (D) Pathological stage. (E) Histological grade. (F) N stage.

To investigate the relationship between *RCN1* and the clinicopathological characteristics of ESCC patients, we analyzed the clinical data of ESCC patients in the GSE53625 dataset. As shown in Fig 3, the expression level of *RCN1* mRNA was not significantly correlated with age, gander, T stage, or pathological stage (all *p* > 0.05). However, the increase in *RCN1* mRNA expression was significantly associated with the N stage progression (N0 vs. N3, N2 vs. N3) and histological grade (G1 vs. G3, G2 vs. G3) (all *p* < 0.05). Subsequently, we evaluated the relationship between the protein level of RCN1 and the clinicopathological characteristics of ESCC patients. We analyzed the clinical data of 80 ESCC patients collected from the clinic; according to the median value of the immunohistochemical IRS score, patients with an IRS score ≤6 were classified into the low-expression group, while those with an IRS score >6 were classified into the high-expression group. The results also showed that high expression of the RCN1 protein was significantly associated with lymph node metastasis in ESCC patients (*p* = 0.026) and advanced pathological stage (*p* = 0.045) (Table 1). Based on the above results, we hypothesize that *RCN1* may participate in the progression of ESCC by promoting tumor metastasis.

**Table 1:** Correlation analysis between *RCN1* expression and clinicopathological characteristics in esophageal squamous cell carcinoma (ESCC) patients.

Variables	All cases	Low expression of *RCN1*	High expression of *RCN1*	*p* value
Total	80	40	40	
Gender				0.822
Male	45	23	22	
Female	35	17	18	
Age (years)				0.204
≥60	21	13	8	
<60	59	27	32	
Cigarettes				0.104
Yes	29	18	11	
No	51	22	29	
Alcohol				0.626
Yes	24	13	11	
No	56	27	29	
Pathological Stage				0.025*
Ia+Ib+IIa+IIb	44	27	17	
IIIa+IIIb+IVa+IVb	36	13	23	
Tumor Location				0.363
Upper	12	5	7	
Middle	52	29	23	
Low	16	6	10	
Histological Grade				0.672
G1	29	13	16	
G2	47	25	22	
G3	4	2	2	
T Stage				1
T1+T2	36	18	18	
T3+T4	44	22	22	
N Stage				0.014*
N0	43	27	16	
N1+N2+N3	37	13	24	
M Stage				1
M0	78	39	39	
M1	2	1	1	

*p < 0.05 is significant.

### Knockdown of RCN1 inhibits migration, invasion, and EMT of ESCC cells

**Fig 4:**
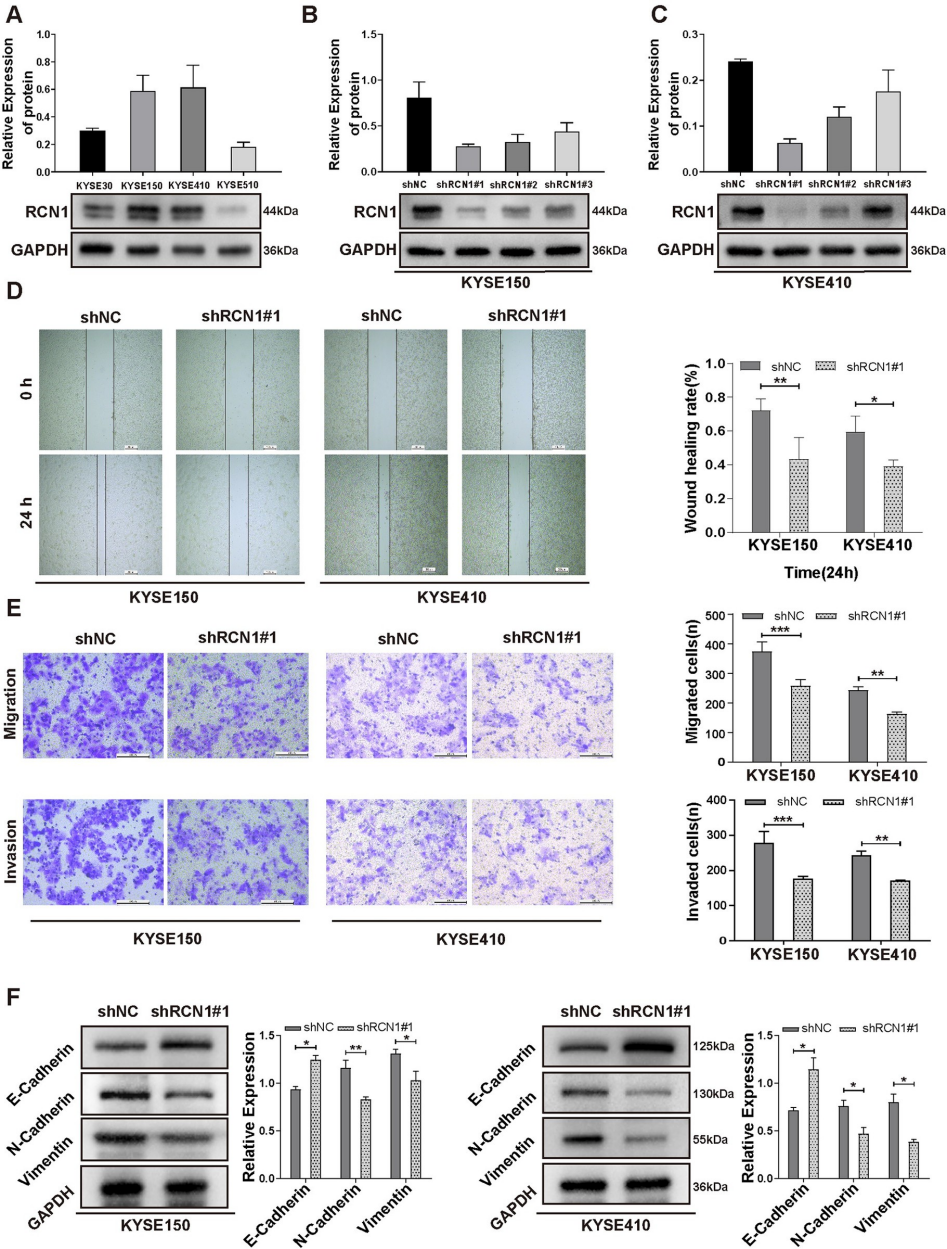
The biological role of *RCN1* in the migration, invasion, and epithelial–mesenchymal transition (EMT) of esophageal squamous cell carcinoma (ESCC) cells. (A) Protein expression of RCN1 in four ESCC cell lines (KYSE30, KYSE150, KYSE450, and KYSE510) was analyzed by western blotting. (B and C) The protein expression of RCN1 in the KYSE150 and KYSE410 cell lines was decreased by transfection with shRNAs (shRCN1#1, shRCN1#2, and shRCN1#3). (D) The migration ability of KYSE150 and KYSE410 cells transfected with shRCN1#1 was assessed by scratch experiments. (E) Migration and invasion abilities of KYSE150 and KYSE410 cells transfected with shRCN1#1 were assessed in transwell assays. (F) The expression levels of EMT-related markers (E-cadherin, N-cadherin, and vimentin) in KYSE150 and KYSE410 cells transfected with shRCN1#1 were detected by western blotting. *p* *<0.05, **p <0.01, ***p <0.001.

To investigate the biological function of *RCN1* in ESCC, we first evaluated the protein expression levels of RCN1 in ESCC cell lines (KYSE30, KYSE150, KYSE410, and KYSE510) by western blotting analyses. The results showed that KYSE150 and KYSE410 cells had higher expression levels of RCN1 (Fig 4A). Subsequently, we transfected three types of *RCN1* shRNAs (shRCN1#1, shRCN1#2, and shRCN1#3) into KYSE150 and KYSE410 cells and found that sh-RCN1#1 had the highest knockdown efficiency in both cell lines by western blot (Fig 4B and 4C). Therefore, sh-RCN1#1 was used for subsequent studies. Previously, our results suggested that *RCN1* was associated with tumor metastasis. To confirm this at the cellular level, we conducted cell scratch and transwell assays. The results showed that knockdown of *RCN1* significantly inhibited the migration and invasion abilities of KYSE150 and KYSE410 cells in both the scratch and transwell assays (Fig 4D and 4E). Previous studies have shown that EMT plays an important role in the invasion and metastasis of tumor cells [21]. To confirm whether RCN1 is involved in the regulation of EMT in ESCC cells, we analyzed the expression of an epithelial marker (E-cadherin) and mesenchymal markers (N-cadherin, vimentin) by western blotting. The results showed that the knockdown of *RCN1* in KYSE150 and KYSE410 cells significantly increased the expression of E-cadherin and decreased the expression of N-cadherin and Vimentin (Fig 4F). In conclusion, knockdown of *RCN1* inhibits the migration and invasion of ESCC cells by suppressing EMT.

### Knockdown of RCN1 promotes apoptosis of ESCC cells

**Fig 5:**
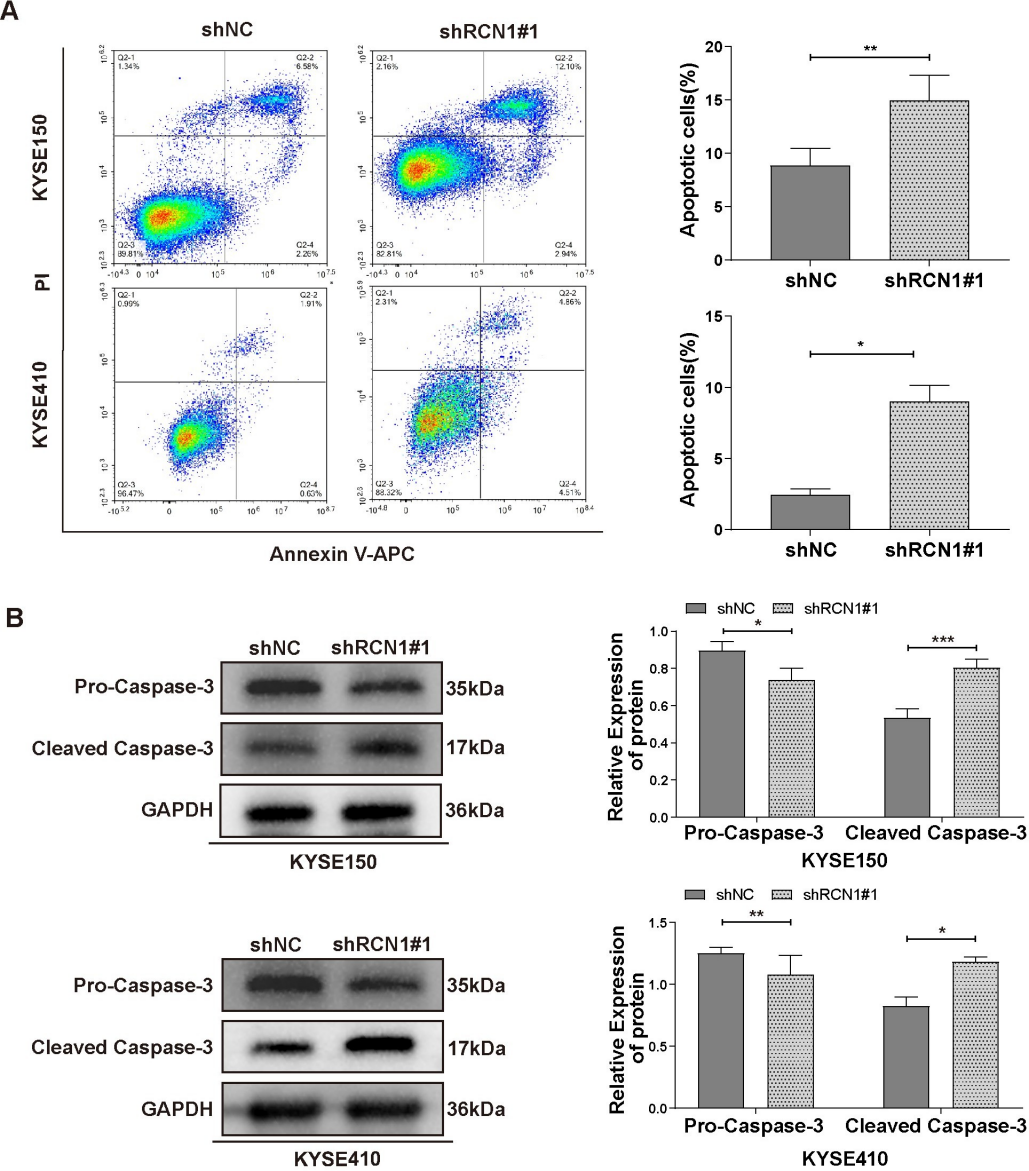
The effect of *RCN1* knockdown on apoptosis in ESCC cells. (A) The effect of *RCN1* knockdown on the apoptosis of KYSE150 and KYSE410 cells was examined using flow cytometry. (B) The effect of *RCN1* knockdown on pro-caspase-3 and cleaved caspase-3 protein expression in KYSE150 and KYSE410 cells was examined using western blotting. *p <0.05, **p <0.01.

Next, we investigated the role of *RCN1* in ESCC cell apoptosis. Flow cytometry analysis revealed that knocking down *RCN1* significantly increased the apoptosis of KYSE150 and KYSE410 cells (Fig 5A). In addition, western blot analysis revealed a decrease in pro-caspase-3 and an increase in cleaved caspase-3 in two cell lines transfected with shRCN1#1 (Fig 5B). These results suggest that knockdown of *RCN1* promotes ESCC cell apoptosis.

### Knockdown of RCN1 in ESCC cells inhibits M2 macrophage polarization

**Fig 6:**
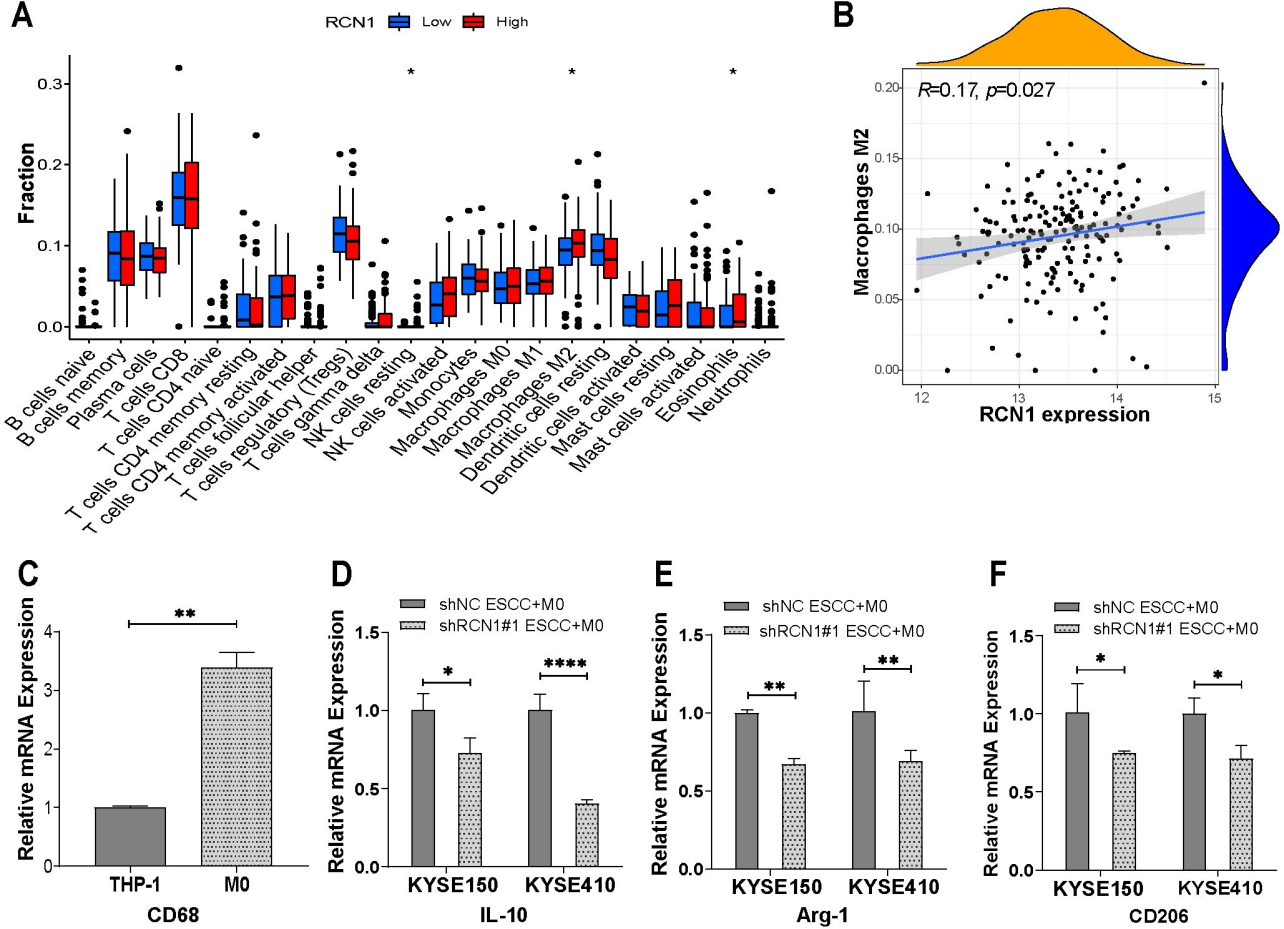
The relationship between *RCN1* expression and M2 macrophages. (A) Differential analysis of tumor-infiltrating immune cells between the high and low *RCN1* expression groups. (B) Correlation of *RCN1* expression with M2 macrophages. (C) Differential expression of *CD68* mRNA between M0 macrophages differentiated from THP-1 cells. (D-F) The mRNA expression levels of M2 macrophage markers (*IL10*, *Arg-1*, and *CD206*) after coculture of M0 macrophages with ESCC cells. *p <0.05, **p* *<0.01.

To investigate the role of *RCN1* in the tumor microenvironment, we first analyzed the effect of *RCN1* expression on tumor-infiltrating immune cells using the GSE53625 dataset. As shown in Fig 6A, the high-expression group exhibited a significant increase in M2 macrophages, resting natural killer (NK) cells, and eosinophils, while there was also a positive correlation between *RCN1* expression and M2 macrophages, as demonstrated in Fig 6B. Previous studies have shown that M2 macrophages can promote the growth, migration, and invasion ability of tumor cells and inhibit immune cell-mediated killing of tumor cells, leading to tumor progression [22]. To confirm that *RCN1* can regulate M2 macrophage polarization, we first induced suspension THP-1 cells to adhere to the culture dish as M0 macrophages using PMA. qRT‒PCR results showed a significant upregulation of mRNA levels for the macrophage marker *CD68* (Fig 6C). Subsequently, KYSE150 and KYSE410 cells with *RCN1* knockdown were cocultured with M0 macrophages, and markers (*IL-10*, *Arg-1*, and *CD206*) of M2 macrophages were detected using qRT‒PCR. The results showed a significant downregulation of *IL-10*, *Arg-1*, and *CD206* expression in the cocultured macrophages after knockdown of *RCN1* in KYSE150 and KYSE410 cells (Fig 6D–6F). These results suggest that knocking down *RCN1* inhibits the M2 polarization of macrophages.

## Discussion

*RCN1* encodes a highly conserved calcium-binding protein belonging to the CREC family of proteins. Recent studies have shown that *RCN1* plays a tumor-promoting role in tumor progression [9, 10, 12, 23]; however, the role of *RCN1* in ESCC has not yet been studied. Our study explored the expression pattern, clinical value, and biological role of *RCN1* in ESCC and the regulatory role of *RCN1* in macrophage polarization.

First, we confirmed that *RCN1* is highly expressed in ESCC tissues at both the mRNA and protein levels using the GSE53625 dataset and clinical samples. Previous studies have also reported that *RCN1* is highly expressed in lung cancer [12], liver cancer [10], laryngeal carcinoma [24], renal cell carcinoma [25], and prostate cancer [13], which is consistent with our research findings. The results of the ROC curve analysis indicated that *RCN1* could serve as a potential diagnostic biomarker. Moreover, our study found that high expression of *RCN1* is associated with a poor prognosis in patients, rendering them more likely to develop lymph node metastasis. Therefore, we speculate that *RCN1* may promote the progression ESCC by increasing the invasiveness of ESCC cells. Subsequent *in vitro* experiments also confirmed that knocking down *RCN1* inhibited the migration and invasion ability of ESCC cells. An increasing number of studies have confirmed the close correlation between acquisition of the migration and invasion abilities of tumor cells and EMT [26, 27]. Our research results indicate that knockdown of *RCN1* can inhibit EMT in ESCC cells, which is consistent with the research results in liver cancer cells [10].

Previous studies have found that calcium influx can regulate cell apoptosis [28]; RCN1 is a calcium-binding protein that participates in regulating the transport of calcium ions. Therefore, we speculate that *RCN1* can regulate cell apoptosis. Subsequently, our experimental results also confirmed that knockdown of *RCN1* can promote tumor cell apoptosis. Similarly, it has been confirmed in prostate cancer [13] and nasopharyngeal carcinoma [29] that *RCN1* can promote tumor progression by inhibiting cell apoptosis.

TAMs have been widely recognized to be closely related to tumor development and metastasis. TAMs are highly plastic, and M1 macrophages with antitumor effects and M2 macrophages with pro-tumor effects can convert into each other under the stimulation of the tumor microenvironment. Multiple studies have shown that M2 macrophages can promote tumor growth, invasion, and metastasis [30, 31]. Therefore, reducing the infiltration or polarization of M2 macrophages is a very promising therapeutic approach. Our analysis of tumor-infiltrating immune cells found that the expression of M2 macrophages was positively correlated with *RCN1*. Subsequently, through co-culturing M0 macrophages with ESCC cells, we found that knocking down *RCN1* resulted in a significant decrease in M2 macrophage markers. These results suggest that knocking down *RCN1* can inhibit M2 polarization. Recently, a study in oral squamous cell carcinoma also found that *RCN1* can regulate M2 macrophage polarization [32]. Previous studies have shown that *RCN1* can downregulate the expression of the *PTEN* gene [13]. Multiple studies have further confirmed that *PTEN* serves as a regulatory factor in TAMs, and inhibiting PTEN can promote M2 macrophage polarization by activating the PI3K/AKT signaling pathway [33–35]. Therefore, we speculate that *RCN1* may regulate M2 macrophage polarization through the PTEN/PI3K/AKT signaling pathway. We plan to conduct further research to validate our hypothesis. Overall, these findings suggest that *RCN1* has multiple pro-tumorigenic roles and is a potential therapeutic target for ESCC.

Despite our findings, there are certain limitations and shortcomings in our study. First, it is necessary to collect specimens from multiple medical centers to evaluate the prognostic and diagnostic value of *RCN1* in patients with ESCC. Second, it is necessary to confirm the regulatory role of *RCN1* in the biological behavior of ESCC cells and macrophage polarization by overexpressing *RCN1*. Third, more experiments are needed to verify the regulatory role of *RCN1* in macrophage polarization, and the specific regulatory mechanisms need to be further elucidated.

## Conclusion

This study confirmed that *RCN1* is highly expressed in ESCC tissues and is associated with lymphatic metastasis and poor prognosis, indicating that *RCN1* has the potential to serve as a diagnostic and prognostic marker in ESCC patients. Further research revealed that knocking down *RCN1* can inhibit ESCC cell migration, invasion, and EMT; promote ESCC cell apoptosis; and suppress M2 macrophage polarization. These results suggest that *RCN1* may become a new therapeutic target for ESCC treatment.

## Supporting information

S1 Table Clinicopathological parameters of ESCC cohort in Affiliated Hospital of North Sichuan Medical College. (DOCX)

S2 Table Primers used for qRT-PCR. (DOCX)

S3 Table Information of all antibodies used in the experiments. (DOCX)

S1 Raw image (PDF)
