# Supplementary material for: Downregulation of RCN1 inhibits esophageal squamous cell carcinoma progression and M2 macrophage polarization
Source: PLoS One. 2024 May 7;19(5):e0302780. doi: 10.1371/journal.pone.0302780 (PMC11075840; doi:10.1371/journal.pone.0302780)
Supplement: S1 Table — (DOCX) [file pone.0302780.s001.docx]

| **Table 1** | | | | | | | | | | | | |
| --- | --- | --- | --- | --- | --- | --- | --- | --- | --- | --- | --- | --- |
| **Clinicopathological parameters of ESCC cohort in Affiliated Hospital of North Sichuan Medical College.** | | | | | | | | | | | | |
| **Patient NO.** | **Gender** | **Age(y)** | **History of smoking** | **History of alcohol** | **Tumour Location** | **Histological Grade** | **T Stage** | **N Stage** | **M Stage** | **Pathological Stage** | **IRS score of RCN1 in tumor tissues** | **IRS score of RCN1 in paraneoplastic tissues** |
| 1 | Male | 71 | No | No | Middle | G1 | T1 | N0 | M0 | IA | 7.00 | 5.33 |
| 2 | Female | 81 | No | No | Middle | G2 | T1 | N0 | M0 | IB | 4.67 | 2.00 |
| 3 | Male | 62 | No | No | Middle | G3 | T2 | N0 | M0 | IIA | 9.00 | 2.00 |
| 4 | Male | 62 | No | No | Low | G1 | T3 | N1 | M0 | IIIB | 6.33 | 3.33 |
| 5 | Female | 67 | No | No | Middle | G1 | T4 | N2 | M0 | IVA | 4.00 | 3.33 |
| 6 | Male | 61 | Yes | Yes | Middle | G2 | T1 | N0 | M0 | IB | 6.33 | 3.33 |
| 7 | Male | 67 | Yes | Yes | Low | G1 | T3 | N1 | M0 | IIIB | 8.00 | 2.00 |
| 8 | Male | 67 | Yes | Yes | Middle | G2 | T1 | N0 | M0 | IB | 8.00 | 2.00 |
| 9 | Male | 50 | Yes | Yes | Middle | G2 | T2 | N0 | M0 | IIA | 4.00 | 2.00 |
| 10 | Female | 71 | No | No | Low | G2 | T1 | N0 | M0 | IB | 8.00 | 2.00 |
| 11 | Male | 72 | Yes | No | Middle | G2 | T1 | N1 | M0 | IIB | 9.00 | 2.00 |
| 12 | Female | 51 | No | No | Middle | G1 | T1 | N0 | M0 | IA | 4.67 | 2.00 |
| 13 | Male | 55 | Yes | No | Middle | G1 | T3 | N0 | M0 | IIA | 3.33 | 6.00 |
| 14 | Male | 62 | Yes | Yes | Middle | G1 | T4 | N1 | M0 | IVA | 9.00 | 2.00 |
| 15 | Female | 56 | No | No | Middle lower | G2 | T3 | N1 | M0 | IIIB | 8.00 | 6.00 |
| 16 | Female | 57 | No | No | Middle lower | G2 | T3 | N1 | M0 | IIIB | 8.00 | 2.00 |
| 17 | Male | 71 | No | Yes | Middle | G2 | T3 | N1 | M0 | IIIB | 8.00 | 2.33 |
| 18 | Male | 63 | Yes | Yes | Middle | G1 | T1 | N0 | M0 | IA | 6.00 | 2.00 |
| 19 | Male | 52 | Yes | Yes | Middle | G1 | T2 | N0 | M0 | IB | 5.33 | 5.33 |
| 20 | Female | 74 | No | No | Low | G2 | T3 | N3 | M0 | IVA | 6.00 | 5.00 |
| 21 | Male | 50 | Yes | Yes | Low | G2 | T3 | N2 | M0 | IIIB | 6.00 | 3.67 |
| 22 | Female | 65 | No | No | Middle | G1 | T2 | N0 | M0 | IB | 7.33 | 2.00 |
| 23 | Male | 77 | No | Yes | Low | G2 | T3 | N2 | M0 | IIIB | 7.33 | 6.00 |
| 24 | Female | 65 | No | No | Middle | G1 | T3 | N2 | M0 | IIIB | 4.00 | 2.00 |
| 25 | Female | 57 | No | No | Middle | G1 | T1 | N0 | M0 | IA | 9.00 | 6.00 |
| 26 | Female | 53 | No | No | Middle | G2 | T2 | N0 | M0 | IIA | 5.33 | 2.00 |
| 27 | Male | 56 | Yes | Yes | Middle | G2 | T1 | N0 | M0 | IB | 5.33 | 2.00 |
| 28 | Male | 68 | Yes | Yes | Middle | G2 | T1 | N0 | M0 | IB | 5.33 | 2.00 |
| 29 | Male | 65 | Yes | Yes | Upper | G3 | T2 | N0 | M0 | IIA | 5.33 | 2.00 |
| 30 | Male | 77 | Yes | No | Middle | G1 | T3 | N0 | M0 | IIA | 5.33 | 2.00 |
| 31 | Male | 64 | No | Yes | Middle | G2 | T3 | N0 | M0 | IIB | 5.33 | 2.00 |
| 32 | Male | 51 | Yes | No | Middle | G2 | T3 | N3 | M1 | IVB | 4.00 | 2.67 |
| 33 | Female | 65 | No | No | Low | G2 | T2 | N0 | M0 | IIA | 4.00 | 2.67 |
| 34 | Male | 66 | Yes | Yes | Low | G2 | T4 | N1 | M0 | IIIB | 9.00 | 2.00 |
| 35 | Male | 56 | Yes | Yes | Low | G2 | T2 | N1 | M0 | IIIA | 7.00 | 6.00 |
| 36 | Male | 54 | No | No | Middle | G1 | T3 | N0 | M0 | IIA | 8.00 | 2.00 |
| 37 | Male | 77 | Yes | No | Upper | G2 | T4 | N2 | M0 | IVA | 6.00 | 2.33 |
| 38 | Male | 61 | Yes | No | Middle | G2 | T2 | N0 | M0 | IIA | 4.67 | 2.00 |
| 39 | Male | 70 | No | No | Upper | G2 | T3 | N1 | M0 | IIIB | 8.00 | 2.67 |
| 40 | Female | 61 | No | No | Upper | G2 | T2 | N0 | M0 | IIA | 9.00 | 2.00 |
| 41 | Female | 64 | No | No | Middle | G2 | T3 | N1 | M0 | IIIB | 7.00 | 2.00 |
| 42 | Female | 64 | No | No | Middle | G1 | T4 | N3 | M0 | IVA | 7.33 | 6.00 |
| 43 | Male | 65 | No | No | Middle | G2 | T3 | N1 | M0 | IIIB | 4.00 | 3.33 |
| 44 | Male | 64 | Yes | No | Middle | G2 | T3 | N0 | M0 | IIB | 6.00 | 2.00 |
| 45 | Male | 65 | No | No | Upper | G1 | T3 | N1 | M0 | IIIB | 7.00 | 2.00 |
| 46 | Male | 72 | No | No | Middle | G2 | T2 | N1 | M0 | IIIA | 7.33 | 2.00 |
| 47 | Female | 50 | No | No | Middle | G1 | T3 | N1 | M0 | IIIB | 9.00 | 4.00 |
| 48 | Female | 53 | No | No | Middle | G2 | T1 | N0 | M0 | IB | 5.33 | 2.67 |
| 49 | Male | 70 | Yes | Yes | Middle | G2 | T1 | N0 | M0 | IB | 9.00 | 2.67 |
| 50 | Male | 63 | No | No | Upper | G2 | T1 | N0 | M0 | IB | 9.00 | 6.00 |
| 51 | Male | 71 | Yes | Yes | Middle | G1 | T3 | N1 | M0 | IIIB | 9.00 | 2.00 |
| 52 | Male | 71 | No | No | Middle | G2 | T3 | N0 | M0 | IIB | 6.33 | 2.33 |
| 53 | Male | 63 | No | No | Middle | G1 | T2 | N0 | M0 | IB | 7.00 | 2.00 |
| 54 | Male | 60 | Yes | Yes | Low | G2 | T3 | N2 | M0 | IIIB | 6.33 | 2.00 |
| 55 | Male | 69 | Yes | No | Low | G1 | T2 | N0 | M0 | IB | 9.00 | 4.67 |
| 56 | Female | 62 | No | No | Middle | G1 | T3 | N0 | M0 | IIA | 5.33 | 2.33 |
| 57 | Male | 71 | No | No | Middle | G2 | T2 | N1 | M0 | IIIA | 8.00 | 2.00 |
| 58 | Female | 76 | No | No | Middle | G3 | T2 | N0 | M0 | IIA | 5.33 | 1.67 |
| 59 | Male | 67 | Yes | Yes | Middle | G1 | T4 | N1 | M0 | IIIB | 6.33 | 1.67 |
| 60 | Female | 57 | No | No | Upper | G2 | T1 | N0 | M0 | IB | 4.00 | 2.00 |
| 61 | Male | 71 | Yes | Yes | Middle | G2 | T4 | N1 | M0 | IIIB | 9.00 | 2.00 |
| 62 | Male | 62 | No | No | Upper | G1 | T1 | N0 | M0 | IB | 7.00 | 2.00 |
| 63 | Male | 72 | No | No | Middle | G1 | T3 | N0 | M0 | IIA | 6.00 | 2.00 |
| 64 | Male | 75 | Yes | Yes | Low | G1 | T2 | N0 | M0 | IB | 5.33 | 2.00 |
| 65 | Male | 71 | No | No | Middle | G2 | T1 | N0 | M0 | IB | 4.67 | 1.67 |
| 66 | Female | 49 | No | No | Middle | G2 | T2 | N0 | M0 | IIA | 6.33 | 6.00 |
| 67 | Female | 57 | No | No | Middle | G2 | T3 | N1 | M0 | IIIB | 9.00 | 2.00 |
| 68 | Female | 64 | No | No | Low | G2 | T2 | N0 | M0 | IIA | 9.00 | 2.00 |
| 69 | Female | 70 | No | No | Low | G1 | T3 | N0 | M0 | IIA | 9.00 | 4.00 |
| 70 | Female | 76 | No | No | Upper | G3 | T3 | N2 | M0 | IIIB | 9.00 | 2.00 |
| 71 | Female | 65 | No | No | Upper | G2 | T3 | N0 | M0 | IIB | 6.33 | 2.00 |
| 72 | Female | 69 | No | No | Middle | G2 | T2 | N0 | M0 | IIA | 9.00 | 3.33 |
| 73 | Female | 65 | No | No | Low | G2 | T4 | N3 | M0 | IVA | 7.00 | 3.33 |
| 74 | Female | 67 | Yes | No | Middle | G2 | T4 | N1 | M0 | IIIB | 6.00 | 2.00 |
| 75 | Female | 65 | Yes | Yes | Middle | G1 | T3 | N1 | M0 | IIIB | 9.00 | 2.33 |
| 76 | Female | 71 | No | No | Upper | G1 | T3 | N1 | M1 | IVB | 8.00 | 5.33 |
| 77 | Female | 73 | No | Yes | Middle | G2 | T3 | N2 | M0 | IIIB | 6.00 | 2.00 |
| 78 | Female | 56 | No | No | Low | G2 | T3 | N1 | M0 | IIIB | 9.00 | 2.67 |
| 79 | Female | 57 | No | No | Middle | G2 | T3 | N1 | M0 | IIIB | 4.67 | 2.67 |
| 80 | Female | 57 | No | No | Upper middle | G1 | T3 | N0 | M0 | IIA | 4.00 | 5.00 |
