# Supplementary material for: Downregulation of RCN1 inhibits esophageal squamous cell carcinoma progression and M2 macrophage polarization
Source: PLoS One. 2024 May 7;19(5):e0302780. doi: 10.1371/journal.pone.0302780 (PMC11075840; doi:10.1371/journal.pone.0302780)
Supplement: S2 Table — (DOCX) [file pone.0302780.s002.docx]

**Supplementary Table 2. Primers used for qRT-PCR.**

| Gene | Forward primer (5ʹ−3ʹ) | Reverse primer (5ʹ−3ʹ) |
| --- | --- | --- |
| *CD206* | TCCGGGTGCTGTTCTCCTA | CCAGTCTGTTTTTGATGGCACT |
| *CD68* | GGAAATGCCACGGTTCATCCA | TGGGGTTCAGTACAGAGATGC |
| *IL10* | GACTTTAAGGGTTACCTGGGTTG | TCACATGCGCCTTGATGTCTG |
| *Arg1* | GTGGAAACTTGCATGGACAAC | AATCCTGGCACATCGGGAATC |
| *GAPDH* | GGAGTCCACTGGCGTCTTCA | GTCATGAGTCCTTCCACGATACC |
