# Supplementary material for: Downregulation of RCN1 inhibits esophageal squamous cell carcinoma progression and M2 macrophage polarization
Source: PLoS One. 2024 May 7;19(5):e0302780. doi: 10.1371/journal.pone.0302780 (PMC11075840; doi:10.1371/journal.pone.0302780)
Supplement: S3 Table — (DOCX) [file pone.0302780.s003.docx]

**Supplementary Table 3. Information of all antibodies used in the experiments.**

| **Antibody** | **Manucacturer** | **Catalog number** | **Dilution ratio** |
| --- | --- | --- | --- |
| **GAPDH** | Hua-Bio | ET1601-4 | 1:80000 |
| **RCN1** | Abcam | ab210404 | 1:2000 |
| **Pro-caspase3** | Abcam | ab184787 | 1:2000 |
| **Cleaved-caspase3** | Abcam | ab184787 | 1:2000 |
| **vimentin** | Fine-Test | FNab09408 | 1:1000 |
| **N-cadherin** | Fine-Test | FNab05570 | 1:1000 |
| **E-cadherin** | Fine-Test | FNab02617 | 1:1000 |
| **HRP-labeled goat anti-rabbit IgG(H+L)** | Fine-Test | FNSA-0004 | 1:5000 |
