# Supplementary material for: Downregulation of RCN1 inhibits esophageal squamous cell carcinoma progression and M2 macrophage polarization
Source: PLoS One. 2024 May 7;19(5):e0302780. doi: 10.1371/journal.pone.0302780 (PMC11075840; doi:10.1371/journal.pone.0302780)

Original images of western blot

Figure 4A

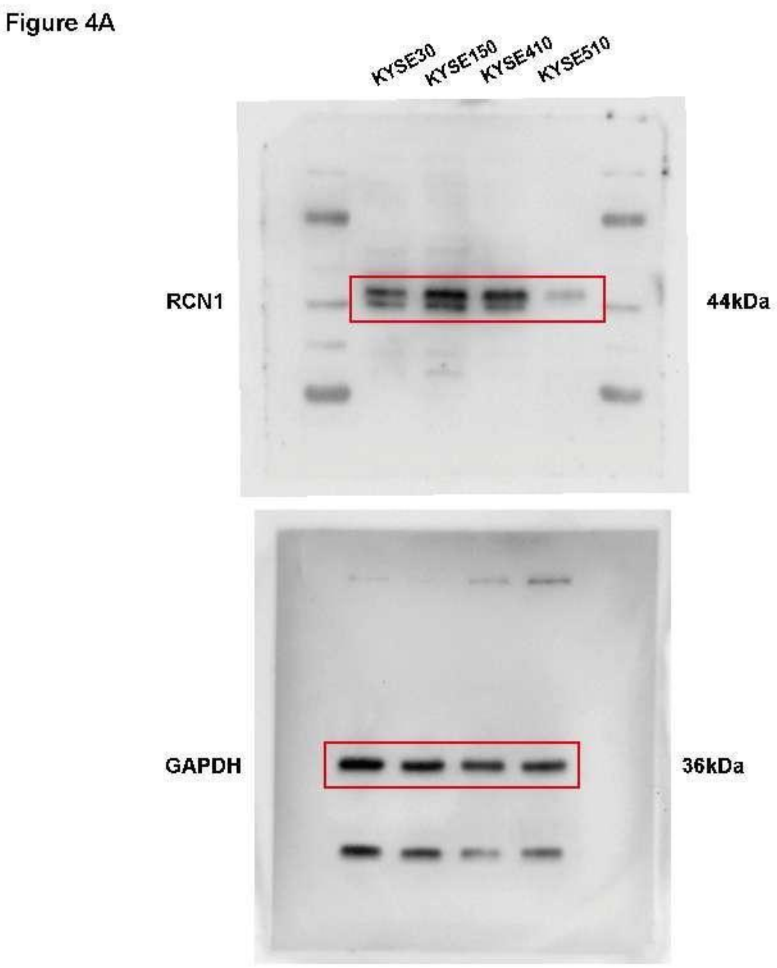

Figure 4B

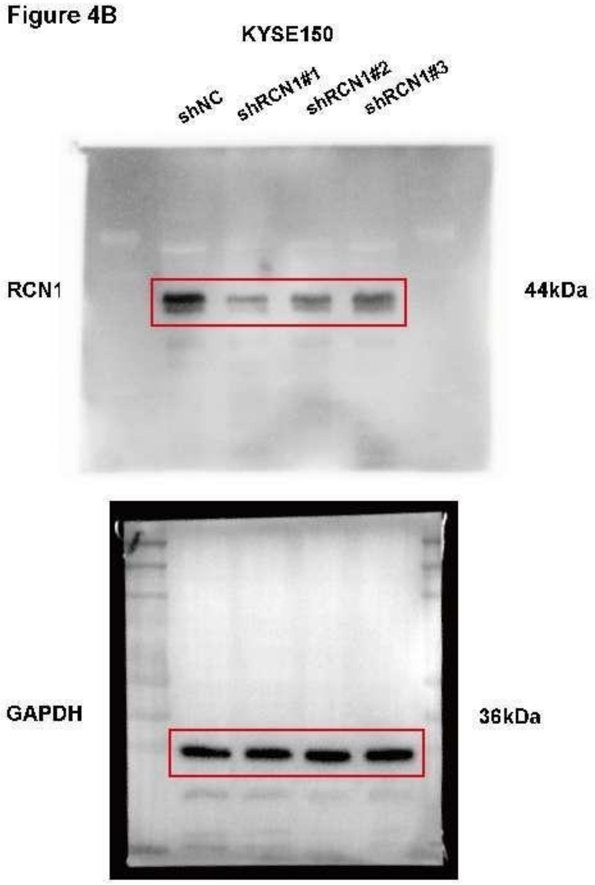

Figure 4C

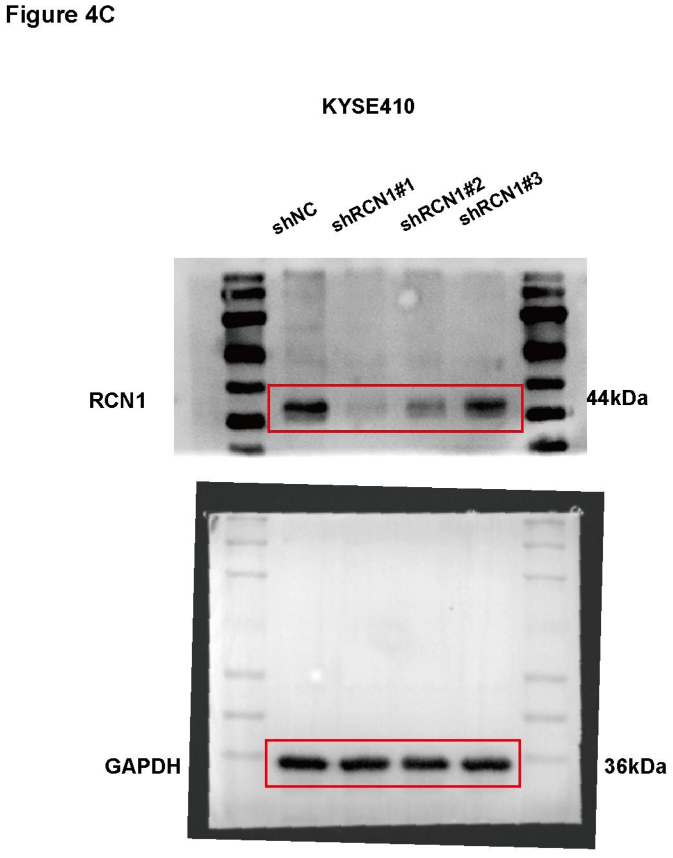

Figure 4F

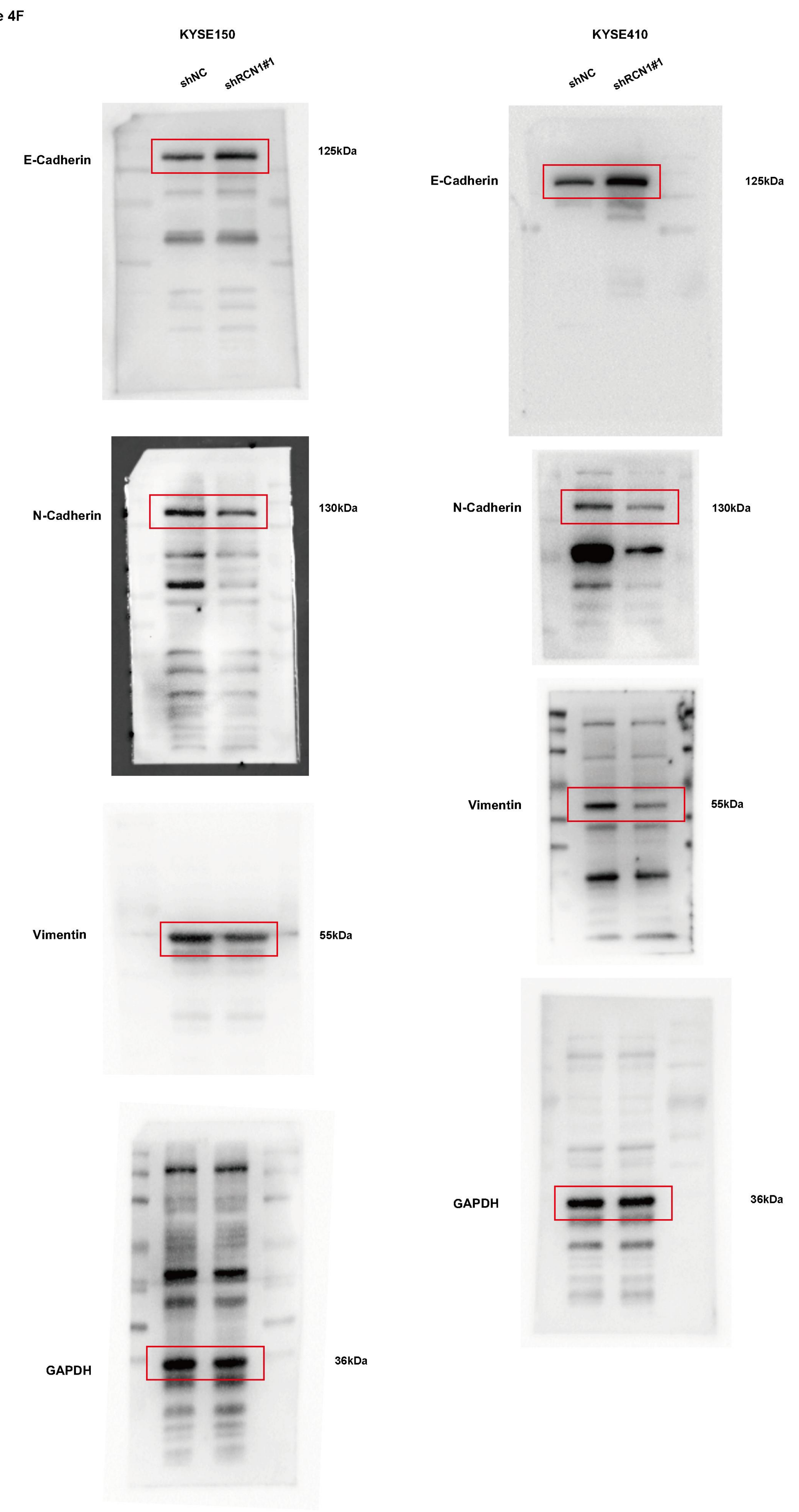

Figure 5B

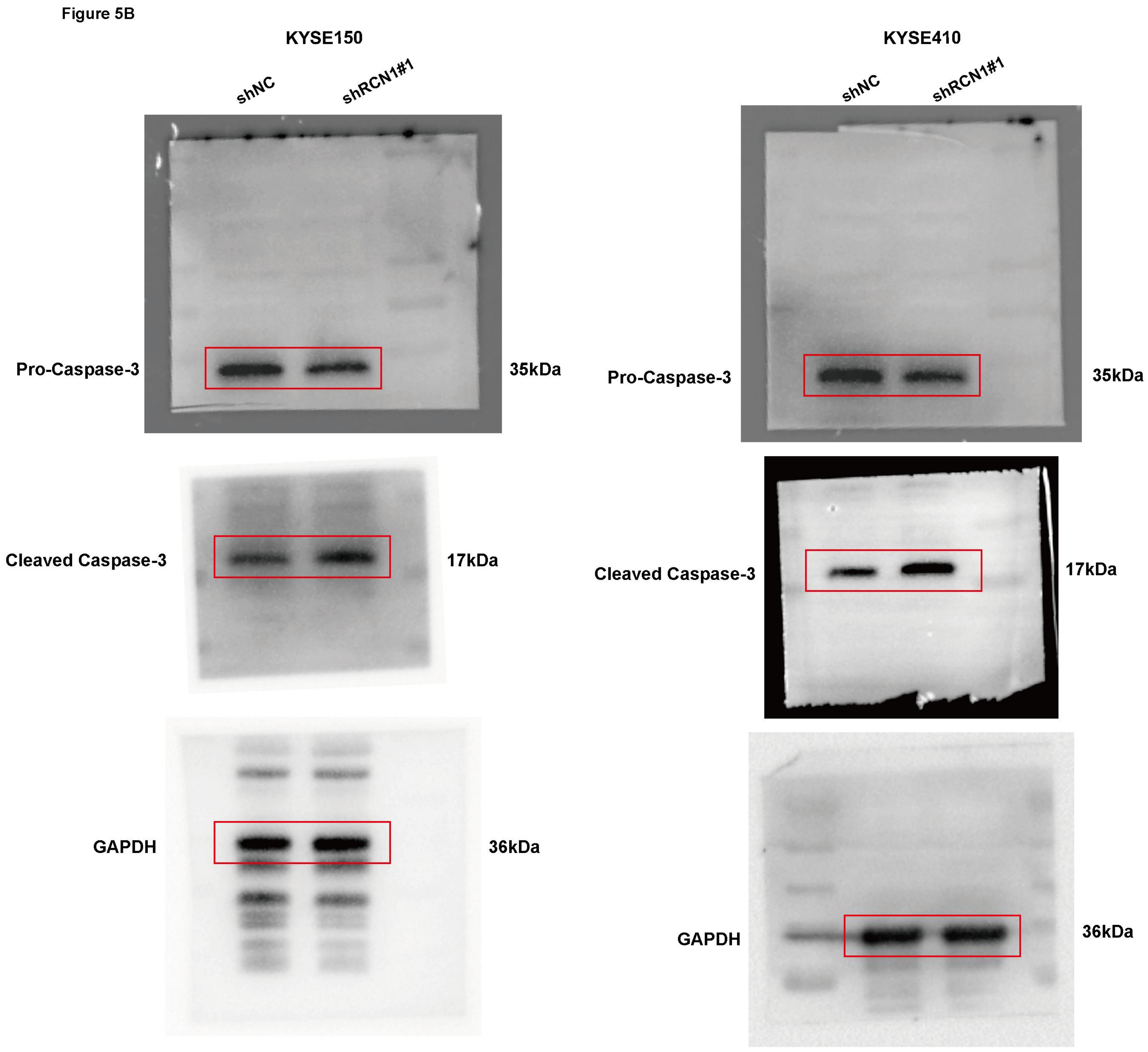

Supplement: S1 Raw image — (PDF) [file pone.0302780.s004.pdf]
